# *Plasmodium cynomolgi* infections in rhesus macaques display clinical and parasitological features pertinent to modelling vivax malaria pathology and relapse infections

**DOI:** 10.1186/s12936-016-1480-6

**Published:** 2016-09-02

**Authors:** Chester Joyner, Alberto Moreno, Esmeralda V. S. Meyer, Monica Cabrera-Mora, The MaHPIC Consortium, Jessica C. Kissinger, John W. Barnwell, Mary R. Galinski

**Affiliations:** 1 International Center for Malaria Research, Education and Development, Emory Vaccine Center, Yerkes National Primate Research Center, Emory University, 954 Gatewood Road, Atlanta, GA 30329 USA; 2 Malaria Branch, Division of Parasitic Diseases and Malaria, Centers for Disease Control and Prevention, Atlanta, GA USA; 3 Division of Infectious Diseases, Department of Medicine, Emory University, Atlanta, GA USA; 4 Department of Genetics, Institute of Bioinformatics, Center for Tropical and Emerging Global Diseases, University of Georgia, Athens, GA USA; 5 Malaria Host–Pathogen Interaction Center, Atlanta, GA USA

**Keywords:** *Plasmodium vivax*, Host-pathogen interactions, Malaria, Non-human primates, Rhesus, Anaemia, Thrombocytopaenia, Systems biology, Animal models

## Abstract

**Background:**

*Plasmodium vivax* infections in humans or in new world monkeys pose research challenges that necessitate the use of alternative model systems. *Plasmodium cynomolg*i is a closely related species that shares genetic and biological characteristics with *P. vivax*, including relapses. Here, the haematological dynamics and clinical presentation of sporozoite-initiated *P. cynomolgi* infections in *Macaca mulatta* (rhesus macaques) are evaluated over a 100-day period.

**Methods:**

Five *M. mulatta* were inoculated with 2000 *P. cynomolgi* B strain sporozoites. Parasitological and haematological data were collected daily to study the clinical presentations of primary infections and relapses. Peripheral blood and bone marrow aspirates were collected at specific time points during infection for future and retrospective systems biology analyses.

**Results:**

Patent infections were observed between days 10 and 12, and the acute, primary infection consisted of parasitaemias ranging from 269,962 to 1,214,842 parasites/µl (4.42–19.5 % parasitaemia). All animals presented with anaemia, ranging from moderate (7–10 g/dl) to severe (<7 g/dl), based on peripheral haemoglobin concentrations. Minimum haemoglobin levels coincided with the clearance of parasites and peripheral reticulocytosis was evident at this time. Mild thrombocytopaenia (<150,000 platelets/µl) was observed in all animals, but unlike haemoglobin, platelets were lowest whenever peripheral parasitaemia peaked. The animals’ conditions were classified as non-severe, severe or lethal (in one case) based upon their clinical presentation. The lethal phenotype presented uniquely with an exceptionally high parasitaemia (19.5 %) and lack of a modest reticulocyte release, which was observed in the other animals prior to acute manifestations. One or two relapses were observed in the four surviving animals, and these were characterized by significantly lower parasitaemias and minimal changes in clinical parameters compared to pre-infection values.

**Conclusions:**

Rhesus macaque infections initiated by *P. cynomolgi* B strain sporozoites recapitulated pathology of human malaria, including anaemia and thrombocytopaenia, with inter-individual differences in disease severity. Importantly, this study provides an in-depth assessment of clinical and parasitological data, and shows that unlike the primary infections, the relapses did not cause clinical malaria. Notably, this body of research has provided experimental plans, large accessible datasets, and blood and bone marrow samples pertinent for ongoing and iterative systems biology investigations.

**Electronic supplementary material:**

The online version of this article (doi:10.1186/s12936-016-1480-6) contains supplementary material, which is available to authorized users.

## Background

Malaria represents a constant burden and public health challenge in approximately 100 countries worldwide [1]. *Plasmodium falciparum* is responsible for the largest number of cases and malaria-associated deaths, with extensive research efforts focused on understanding the basic biology and associated pathogenesis of this parasite species. *Plasmodium vivax* has also been recognized as a major contributor to the global burden of malaria, and numerous recent reports indicate infections can result in complications with lethal outcomes [2–5]. However by comparison, neglect of *P. vivax* is apparent, greatly due to the challenges associated with studying this species, which include the lack of a long-term in vitro culture system or a rodent model that can support the entire life-cycle and re-create critical aspects of *P. vivax* infections and disease as observed in humans [6–9].

*Plasmodium vivax,* as well as *Plasmodium ovale,* differ from the three other human malaria-causing species (*P. falciparum*, *Plasmodium malariae*, *Plasmodium knowlesi*) because they can develop dormant liver-stage forms, known as hypnozoites [10]. Hypnozoites can activate and multiply, causing relapsing blood-stage infections days, months or years after the primary infection. Relapses may result in clinical disease and, importantly, provide the chance for gametocytes to encounter anopheline mosquito vectors and ensure transmission. New insights are needed to treat this liver-stage reservoir to ensure the elimination of relapses and blood-stage infections containing infectious gametocytes. Virtually nothing is known about the biology of relapse infections despite the fact that relapses are thought to be responsible for as high as 96 % of vivax infections in different parts of the world [11]. It is currently unclear how relapses are similar or different from primary infections, from a parasitological and clinical perspective, and the relative contribution of relapses to clinical malaria has been uncertain.

Understanding the course of primary and relapse infections, and specific mechanisms that result in vivax malaria pathogenesis, disease severity and recovery, are important goals. In particular, the identification of mechanisms that function in the peripheral blood and bone marrow resulting in the onset and recovery of malarial anaemia [12, 13] and thrombocytopaenia [14] may aid the search for novel interventions and therapeutic strategies to manage and/or alleviate these common complications. Headway has been made with the study of specimens isolated from human patients living in endemic areas [15, 16]; however, these studies are mostly restricted to one or a few small blood samples at the time of illness and treatment. Furthermore, the analysis and interpretation of these studies can be affected by uncontrollable variables such as diet, medications, transmission characteristics, co-infections, and other maladies. These factors can confound associations of clinical signs and symptoms. Non-human primate animal models can eliminate a number of these concerns, with experimental plans allowing for the study of specific biological, immunological or pathological processes in a controlled, prospective and manipulable environment.

Non-human primate models have contributed to the broad understanding of malaria biology, pathogenesis and immunity with regards to liver-stage and blood-stage infections, and they have also been instrumental for screening vaccine and drug candidates or specific formulations [17–21]. *Plasmodium vivax* can be studied directly in New World monkey species, such as squirrel (*Saimiri boliviensis*) and owl (*Aotus* sp.) monkeys, and these models have been important, for example, in testing vaccine and drug candidates with parasite challenge infections [22, 23] and, recently, to identify and characterize blood-stage proteomes of *P. vivax* infected erythrocytes [24, 25]. However, due to the small size of these animals (~1 kg), which limits the amount of blood (or bone marrow) available for sampling, and the lack of validated reagents available to evaluate host physiological and immunological responses, they are not ideal for extensive hypothesis testing in relation to malaria pathogenesis, immune responses and recovery processes.

*Plasmodium cynomolgi* is a simian malaria parasite of Old World macaques that is genetically closely related to *P. vivax* [26, 27] and shares many biological similarities to the human parasite including the preferential invasion of reticulocytes [28, 29], development of unique infected red blood cell (RBC) structures called caveola-vesicle complexes [30, 31], and critically, the ability to form hypnozoites that can reactivate and cause relapse infections [10, 18, 32, 33]. Macaques are closely related to humans, and a large variety of cross-reactive reagents for assessing host responses have been developed since these monkeys are model organisms for infectious diseases [34]. Young adult macaques (~5 kg or greater) can support longitudinal *Plasmodium* infection studies that require repeated sampling within a short time frame. *Macaca mulatta* (rhesus monkey) and *M. fascicularis* genome sequences [35, 36] and the genome sequence of several strains of *P. cynomolgi* [26], have been characterized in recent years, enabling basic as well as systems biology studies of host-parasite interactions.

Here, comprehensive analyses of sporozoite-initiated, longitudinal infections of *P. cynomolgi* in rhesus macaques are presented with the long-term goal of using this model for systems biology investigations to better understand human malaria pathogenesis, particularly as it pertains to pathophysiological complications observed in sick patients and with regard to the impact of relapses on both the health of individuals and transmission [37]. Previous studies have utilized the *P. cynomolgi*-macaque model to understand the parasite’s basic biology and parasite kinetics, including the study of hypnozoites and relapses [20, 33, 38]. However, an extensive characterization of the clinical and haematological perturbations that occur during the infections has not been reported, particularly on a daily basis.

## Methods

### Experimental design

**Fig. 1:**
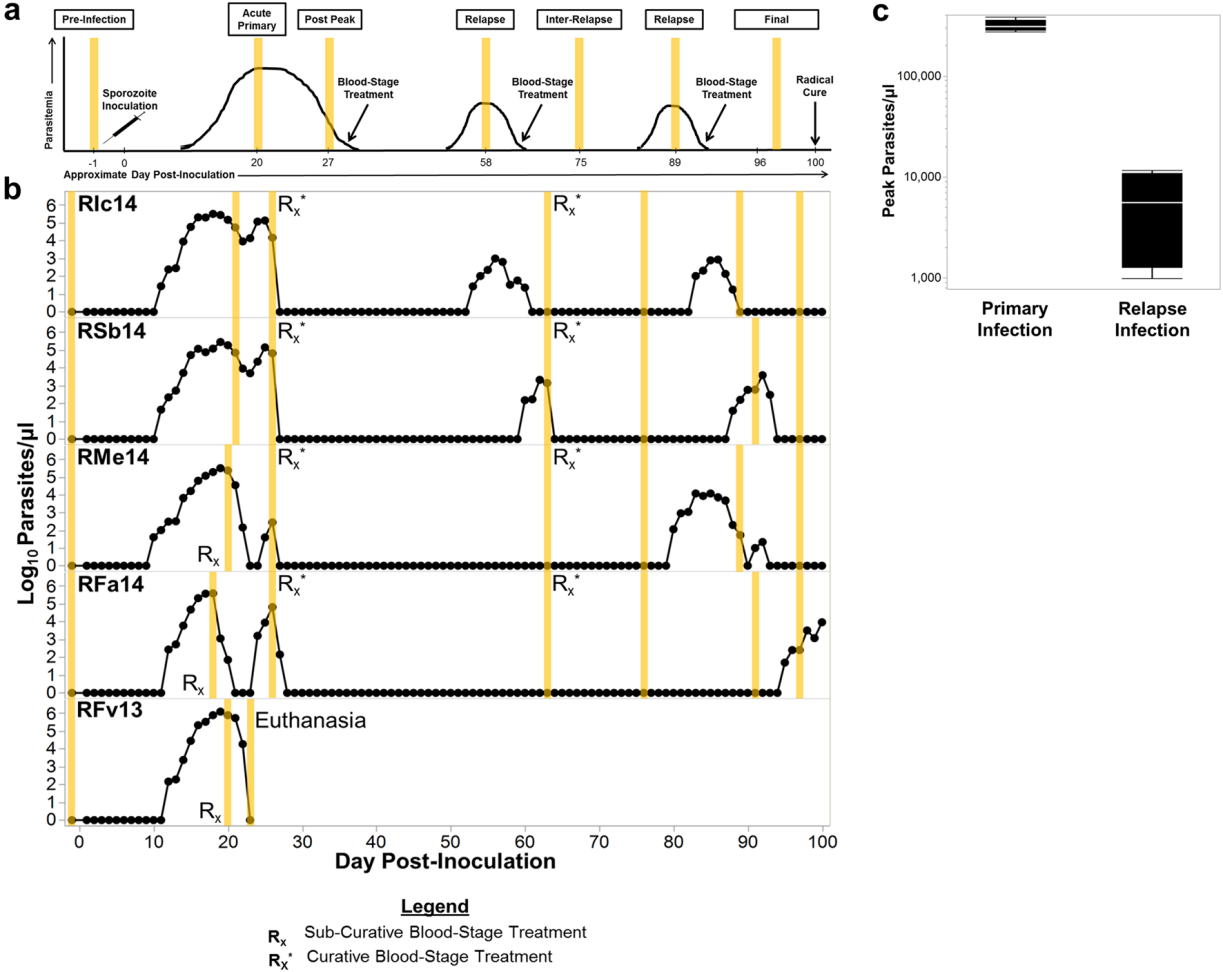
Study design, parasitaemias and treatment regimens of rhesus macaques infected with *Plasmodium cynomolgi* sporozoites. **a** Longitudinal study design to collect whole blood and bone marrow aspirates for current and retrospective analyses at critical infection points as indicated (*yellow*). **b** Log_10_ parasitaemia and treatment regimens for the five rhesus macaques infected with 2000 *P. cynomolgi* B strain sporozoites on day 0 are shown. Whole blood and bone marrow aspirate sample collections are marked in *yellow* for each animal and correspond with critical infection points depicted in *Panel* **a** and summarized in Table 1. Sub-curative and curative blood-stage treatments were administered as needed based on the clinical complications that occurred during the primary infection and to ensure *bona fide* relapses. **c** The mean of the peak parasitaemias for the primary and first relapses of RIc14, RSb14, RMe14, and RFa14 are shown. Statistical significance was assessed by a Wilcoxon-Matched pairs sign rank test; p-values less than 0.05 were considered significant

The basic experimental design of this study is shown in Fig. 1a. The sampling strategy included a single pre-infection and six subsequent blood and bone marrow aspirate specimen collections during the infection at major infection or pathology presentation time points. The criteria for the collection of samples are described in Table 1 and adhered strictly to Institutional Animal Care and Use Committee (IACUC)-approved volume limits of 10 ml/kg/month or 6.6 ml/kg/month if an animal was anaemic. Adjustments were made as to when samples were acquired based on each animal’s parasitological and haematological kinetics. Clinical signs and symptoms observed by trained veterinary staff were also taken into consideration daily. All samples collected for an experimental period satisfied at least one or more of the pre-defined clinical criteria (Table 1). As expected, some individuals required pharmacological and/or clinical support during the acute, primary infection. Blood and bone marrow aspirate specimens were acquired prior to such clinical interventions.

**Table 1:** Criteria for the collection of samples at different experimental periods

Experimental period	Criteria^a^
Pre-infection	Normal motor activity and appetite Haematological parameters within normal range
Acute primary	Prostration, lethargy, low appetite Moderate to severe anaemia, thrombocytopaenia, tachypnea, hyperparasitaemia, impaired consciousness, hyperpyrexia, azotemia, hyponatremia, hyperkalaemia and clinical evidence of activated coagulation (e.g., petechiae)
Post peak	Moderate anaemia, reticulocytemia, control of parasitaemia
Relapse	Low parasitaemia
Inter-relapse	Haematological parameters within normal range, no parasitaemia
Final	Haematological parameters within normal range

^a^The specific criteria and observations at sample collection points during the infection were recorded for each animal based on the clinical assessments made by veterinary staff and investigators

In addition to collecting samples for analysis at major points during the infections, this study was also designed to collect clinical data on a daily basis. The aim of these collections was to develop highly resolved clinical kinetics. To achieve this goal, the animals were accessed daily without sedation for obtaining up to 100 µl of blood through standardized ear-prick procedures, similar to the collection of blood from a human finger-prick. This blood was used to perform complete blood counts (CBCs) as well as enumerate reticulocytes and parasites during the infection.

### Animal use

Five healthy, male malaria-naïve *M. mulatta* (RFa14, RIc14, RMe14, RSb14, RFv13) born and raised at the Yerkes National Primate Research Center (YNPRC), an Association for Assessment and Accreditation of Laboratory Animal Care (AAALAC) international-certified institution, were assigned to this study. The animals were socially housed in pairs at YNPRC during the experiment, and all housing was in compliance with Animal Welfare Act regulations as well as the Guide for the Care and Use of Laboratory Animals. Males were designated to eliminate confounding factors studying anaemia that could be attributed to loss of blood during the female menstrual cycle. An additional rhesus monkey was assigned at the Centers for Disease Control and Prevention (CDC) and utilized for the generation of sporozoites. Standard procedures for monitoring the clinical conditions of the animals, collecting biological samples (venous blood and bone marrow aspirates), and performing mosquito feedings on infected animals to generate infectious salivary gland sporozoites were approved by Emory University’s or the CDC’s IACUC and followed accordingly. All non-human primates used in this study were provided regular environmental enrichment opportunities consisting of daily feeding enrichment, provision of manipulanda, and physical enrichment. Subjects were regularly monitored for any behavioural signs of distress by YNPRC behaviour management personnel. Animals were trained using positive reinforcement to allow blood collections from the ear without sedation.

### Parasite selection

*Plasmodium cynomolgi* B strain was selected for infections because the relapse pattern of this strain had been characterized previously [32], providing confidence that relapse infections would be observed within the 100-day experimental infection period, based on an inoculum size of 2000 freshly dissected salivary gland sporozoites. This strain was also selected because of the availability of its reference genome [26], needed for retrospective system biology studies on the samples that were collected and stored for such purposes.

### Sporozoite generation and inoculation

To generate sporozoites, a donor rhesus macaque was infected with blood-stage parasites that were reconstituted from *P. cynomolgi* B strain, ring-stage cryopreserved stocks maintained by the malaria branch at the CDC. Prior to the start of this study, the identity of these parasites was verified by Illumina 454 sequencing and comparison to the reported genome sequence of Tachibana et al. [26]. Laboratory-bred *Anopheles dirus, Anopheles gambiae* and *Anopheles stephensi* mosquitoes were fed on the donor monkey using standard, IACUC-approved procedures after observance of male and female gametocytes in the blood. Successful infection of the mosquitoes was confirmed within a 7-day period post feeding via midgut dissections to monitor oocyst development and the progression of the infections with sporozoites detected in the salivary glands on days 11 and 12 after mosquitoes fed on infected blood. The salivary glands were freshly dissected from the infected mosquitoes and immediately processed by repeated passage through a 26G needle ten times to release sporozoites in RPMI-1640 culture media supplemented with 10 % fetal calf serum (FCS) on ice. The processed salivary gland and other mosquito debris were allowed to settle by gravity and the sporozoites in the supernatant were washed once by centrifugation at 1000×*g* in sterile culture medium with 10 % FCS. Sporozoite concentrations were determined by counting in a neubauer hemocytometer chamber using a light microscope at 400× magnification. Isolated sporozoites were re-suspended in sterile RPMI 1640 with 10 % FCS, and the macaque cohort infections were initiated by the intravenous inoculation of 2000 sporozoites per animal.

### Clinical monitoring

Blood samples were collected daily from the monkey cohort from day −1 to 100 into EDTA-coated capillary tubes using standardized, IACUC-approved, ear-prick procedures. These samples were used to perform CBCs using an automatic cell analyzer (Beckman AcT-diff; Coulter Corporation) and reticulocyte enumeration using new methylene blue staining of thin blood smears. Clinical data were reviewed with parasitological data daily to monitor the clinical conditions of the animals and determine when specimens should be collected based on the criteria noted in Table 1.

### Parasite enumeration

Parasitaemia counts (parasites/µl) were determined daily via microscopy readings of thick and thin blood films stained with a modified Wrights–Giemsa using enumeration protocols recommended by the World Health Organization (WHO) [39]. Briefly, the number of infected RBCs (iRBC) observed on thick blood smears per 500 leukocytes was recorded, and the parasitaemia determined by multiplying the percentage of parasites out of the total number of leukocytes with the leukocyte concentration obtained from the CBC. When parasitaemia readings were at or greater than 1 %, thin blood smear counts were obtained by counting the number of infected RBCs out of a total of 1000–2000 RBCs. The percentage of infected RBCs was then calculated, and the RBC concentration from the CBC was used to determine the parasitaemia (parasites/µl). The per cent of iRBCs (or per cent parasitaemia) was also calculated by dividing the number of parasites by the RBC count and multiplying by 100. To ensure accuracy, two expert microscopists independently determined the parasitaemias each day. If a significant discrepancy was observed (e.g.  ≥2 standard deviations of the mean) and/or if unexpectedly low or high parasitaemia readings were reported, a third, independent senior expert microscopist was designated to provide an additional parasitaemia reading. All three parasitaemia counts were compared, and the definitive parasitaemia was typically based on the average of the two counts that were the closest together. In other cases, primarily while verifying the presence of relapses with low parasitaemias or after sub-curative blood-stage treatment, the third microscopist’s counts were used as the decisive readings. For relapse verification, the third microscopist counted the number of parasites per 2000 white blood cells (WBCs), which quadrupled the level of sensitivity of the assay.

### Specimen collections

Peripheral blood and bone marrow aspirate samples were collected prior to infection and during the infections (Fig. 1a) for immediate analyses and projected future multi-omic studies (e.g., transcriptomics, proteomic, lipidomics, metabolomics, and immunological profiling) to be performed and integrated with the clinical and parasitological data described here. The animals were anesthetized with ketamine, and blood and bone marrow aspirates were collected from the femoral vein and iliac crest, respectively. Venous and capillary blood samples were collected in EDTA using either BD vacutainer or capillary tubes, respectively, for haematological assays, e.g., CBCs and reticulocyte enumeration, and for parasite enumeration. Blood for clinical chemistry analyses was collected in lithium heparin tubes.

### Clinical definitions

Clinical definitions were based on reference values for rhesus macaques as defined at the YNPRC and other available literature [40]. The normal range of haemoglobin concentration for male rhesus macaques is 12–14 g/deciliter (dl) and anaemia can be similarly defined in humans. Here, anaemia was defined as severe (<7 g/dl), moderate (7–10 g/dl), or mild (10–11 g/dl), based on the haemoglobin nadir, or the lowest level, during the primary infection and relapses. Anaemia was further classified as normocytic (63.7–86.9 femtolitres (fl), microcytic (<63.7 fl) or macrocytic (>86.9 fl) based on the mean corpuscular volume (MCV). Thrombocytopaenia was defined as having a platelet count below 150,000 platelets/µl during either the primary or relapse infections, and severe thrombocytopaenia was defined as having a platelet count of <50,000 platelets/µl.

### Therapeutic interventions

WHO criteria for the management of severe malaria were utilized as a guide to determine when animals were progressing towards severe disease and necessitated clinical and/or therapeutic intervention to ward off terminally severe outcomes [41]. Animals that developed clinical complications associated with severe malaria during the acute infection periods were administered a sub-curative anti-malarial treatment of 1 mg/kg artemether. Additionally, severe anaemia (<7 g/dl) was treated with IV fluid support and whole blood transfusion, also guided by YNPRC veterinarian recommendations and IACUC-approved procedures. After collecting the specimens for the post-peak infection point (Fig. 1a), blood-stage infections were cured with artemether administered at 4 mg/kg on the first day of treatment and 2 mg/kg/day for 7 days thereafter. This ensured that subsequent blood-stage parasitaemias detected in the animals were the result of relapses from the activation of hypnozoites, and not recrudescences of sub-patent, persisting blood-stage forms. Curative blood-stage treatment with artemether was also administered using this same regimen after each relapse, and a curative course of chloroquine (15 mg/kg/day for 3 days administered intramuscularly) and primaquine (1 mg/kg/day for 7 days administered orally) was administered at the end of the entire experiment to treat the blood stages and the hypnozoites, respectively.

### Statistical analysis

Non-parametric statistical approaches were used to assess statistical significance. A Friedman test was performed followed by Dunn’s Multiple Comparison post hoc analysis to identify significant changes in clinical parameters from pre-infection values over time and between pre-, primary, and relapse infection values. Spearman’s non-parametric correlation analysis was performed on clinical parameters using clinical values obtained from the defined experimental periods to ensure that different sample types (i.e., venous blood collected at major infection points *vs* capillary blood collected daily) did not influence associations. A Wilcoxon matched-pairs rank sums test was performed to compare the primary and relapse infection parasitaemias. Both Graphpad Prism Version 6 and JMP Version 12 were used for statistical analysis.

### Data management and release

All samples were tracked with barcodes and a Laboratory Management Information System (LIMS). Data that were manually generated, e.g., clinical assessments, parasitaemias, animal handing, treatment, diet, snacks, and procedures were entered into the LIMS or electronic spreadsheets with double-data entry to increase accuracy. Data fields were constrained with limits or populated with pull-down lists of allowable values to further increase accuracy. All data were then transferred to internal repositories for validation and integration with other data. Standard operating procedures (SOPs) for all methods were collected and archived. Data derived from blood and bone marrow samples were associated with the appropriate animal and experimental metadata via a barcode. All data associated with this study (clinical, experimental, SOPs, and data dictionary to define all terms) were backed-up locally as well as off-site. The dataset supporting the conclusions of this article is included with the article as Additional file 1 and is also being made available via the freely accessible database, PlasmoDB [42], as no archival repositories exist for these types of data. This experiment is known in the deposited files as MaHPIC Experiment 4 (E04).

## Results

### Primary and relapsing parasitological profiles of *P. cynomolgi* B strain in *M. mulatta* during a 100-day experimental infection

Intravenous inoculation of approximately 2000 freshly isolated salivary gland sporozoites produced patent infections within 10 to 12 days (mean ± SE = 11.2 ± 0.37) consistent with previous literature (Fig. 1b; [17, 32]). The maximum parasitaemias during the primary infections ranged from 269,962 to 1,214,842 parasites/µl (4.42–19.5 % iRBC) between days 18–20 post-inoculation (Fig. 1b). RFa14, RMe14 and RFv13 required sub-curative treatment with artemether (1 mg/kg) as noted due to clinical complications (see Table 2). These interventions were implemented to avoid negative outcomes, including possible death. RFa14 and RMe14 developed recrudescent infections 48–72 h later, as anticipated based on prior experience with sub-curative artemether treatments of *P. cynomolgi* and *P. coatneyi* in rhesus macaques at the CDC and the YNPRC [43]. RFv13 subsequently, despite the treatment, developed severe clinical complications, which required this animal to be euthanized, per IACUC guidelines and protocols (Fig. 1b). In summary, this animal presented with acute renal failure. RSb14 and RIc14 did not require sub-curative treatment. These animals were able to control their primary parasitaemia, presumably through an immune mechanism, resulting in an approximate ten-fold reduction in parasite levels from about 300,000 parasites/µl down to 4000–8000 parasites/µl between days 18–23 post-inoculation (Fig. 1b). This reduction in parasitaemia was then followed by a rise again to nearly peak parasitaemia levels, which is characteristic of most *P. cynomolgi* infections (Fig. 1b; [17]).

**Table 2:** Clinical summary and phenotype classification of rhesus macaques infected with *Plasmodium cynomolgi* B strain

	RSb14	RIc14	RFa14	RMe14	RFv13
Anaemia (Degree)	Yes (Moderate)	Yes (Moderate)	Yes (Moderate)	Yes (Severe)	Yes (Severe)
Thrombocytopaenia	Yes	Yes	Yes	Yes	Yes
Unique sign	None	None	Petechiae	None	None
Sub-curative treatment	No	No	Yes	Yes	Yes
Additional clinical interventions	None	None	None	Blood transfusion	Blood transfusion
Clinical phenotype classification	Non-severe	Non-severe	Severe	Severe	Lethal

To distinguish relapses from possible recrudescences of persisting low-level blood-stage parasitaemias, RIc14, RSb14, RFa14, and RMe14 received curative blood-stage treatment with artemether after their primary parasitaemia and illness. Artemether was administered uniformly on day 26 at 4 mg/kg, and then 2 mg/kg/day for 7 days thereafter (Fig. 1b). During the 100-day experiment, RIc14 and RSb14 experienced two relapses, whereas RFa14 and RMe14 had a single relapse (Fig. 1b). RIc14 and RSb14 experienced their first relapse within 19 and 26 days (day 51 and day 58 post-inoculation), respectively, after completion of curative blood-stage treatment on day 32 (Fig. 1b). Contrastingly, RFa14 and RMe14 remained negative by microscopy during this period (Fig. 1b). Curative anti-malarial treatment for blood stages was administered to all animals on day 63, and relapse infections were detected as early as 10 days after the completion of this second curative blood-stage treatment. All animals relapsed prior to the end of the 100-day study, after the second round of curative blood-stage treatment; RIc14 on day 83 and RSb14 on day 88, with RFa14 and RMe14 relapsing for the first times on day 95 and day 80, respectively.

The peak parasitaemia for the first relapse infections ranged from 846 to 11,584 parasites/µl, which was strikingly lower than the primary infection (Fig. 1c). Since the animals as a group experienced either one or more relapses, this comparison was performed using the maximum parasitaemia for the first relapse noted for each individual. This result was not statistically significant (*p* = 0.0625) however, based on the four of five animals remaining in the study. A follow-up study will expand on these numbers. Contrary to a previous report stating that relapse parasitaemias decrease with subsequent relapses [19], a large difference in parasitaemia was not observed between the first and second relapses for RSb14 and RIc14 (Fig. 1b).

### Different degrees of anaemia were observed during the primary blood-stage infections

**Fig. 2:**
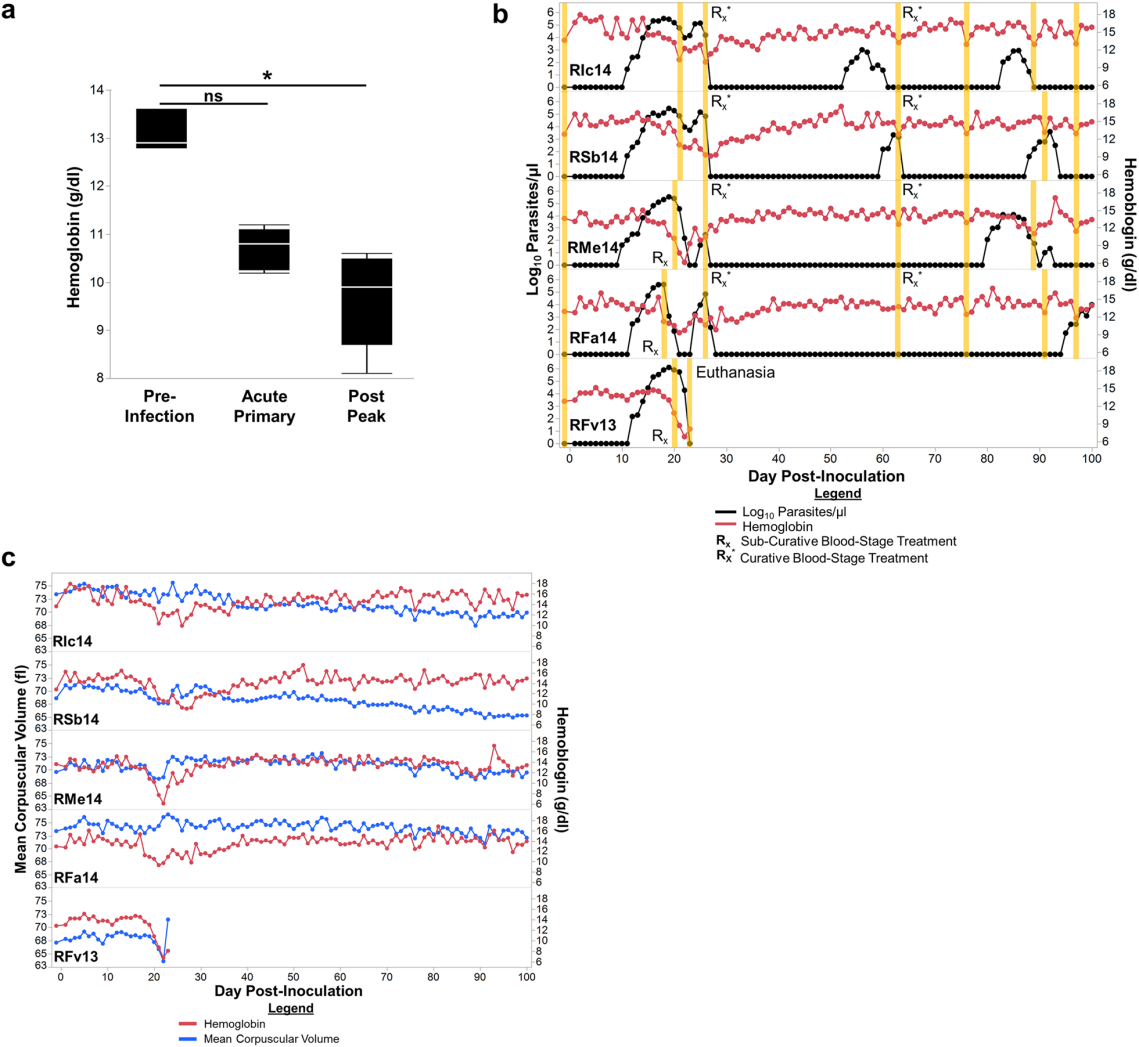
*Plasmodium cynomolgi* B strain infection in rhesus macaques induces moderate to severe anaemia. **a** Haemoglobin concentrations of five rhesus macaques are shown prior to infection, during the acute primary, and after the peak of parasitaemia. **b** Haemoglobin kinetics in relation to parasitaemia and the relationship between haemoglobin and MCV (**c**) for up to 100 days after inoculation are shown. *Orange bars* in Panel B indicate when peripheral blood and bone marrow aspirates were collected for retrospective systems biology analysis. Statistical analysis was performed by a Friedman’s Test with Dunn’s multiple comparison post-hoc for *Panel* **a**; ^*^p < 0.05, *ns* not significant

The primary *P. cynomolgi* B strain blood-stage infection resulted in anaemia in the entire cohort (Fig. 2a, b). Similar to anaemia in humans with malaria, inter-individual differences in anaemia severity were evident and ranged from moderate (7–10 g/dl) to severe (<7 g/dl) (Fig. 2b; Table 2). The anaemia was normocytic (defined as 63.7–86.9 fl) based on MCV values (Fig. 2c). Although parasitaemia and haemoglobin concentration were negatively correlated (Spearman ρ = −0.7288, *p* < 0.0001), a statistically significant decrease in haemoglobin concentration compared to pre-infection values did not coincide with the peak of parasitaemia during the acute, primary phase of the infection (*p* > 0.05; Fig. 2a, b). Rather, the haemoglobin concentrations were significantly lower than pre-infection values approximately 4–7 days after the recognized peak of parasitaemia, a period termed here as the ‘post-peak of parasitaemia’ (Fig. 2a, b). During this period, the macaques were either controlling the infections naturally leading to decreases in parasite numbers, or recovering from the infections after the administration of a sub-curative dose of artemether. Importantly, anaemia to some degree was observed irrespective of the administration of sub-curative treatment (Fig. 2b).

**Fig. 3:**
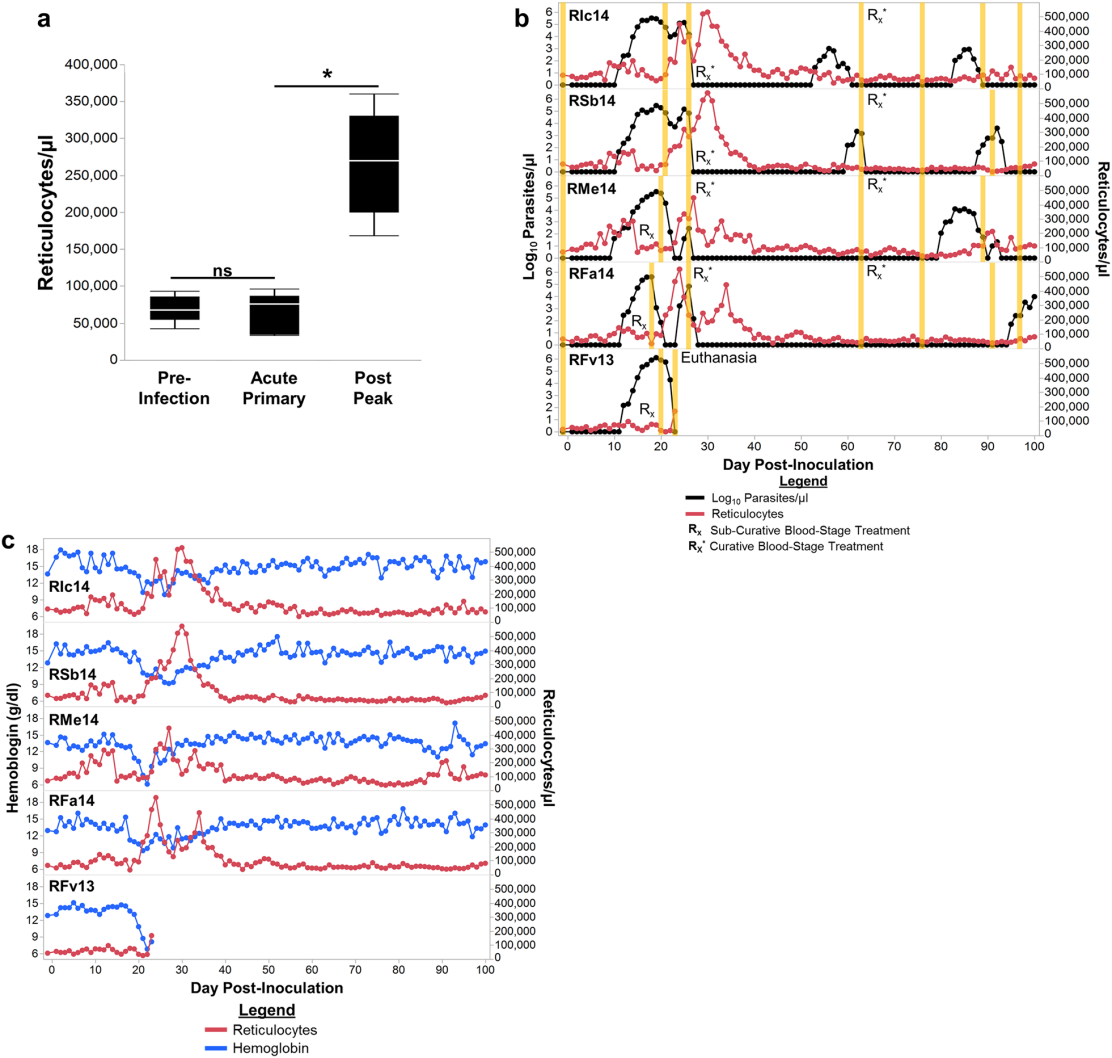
Insufficient erythropoietic output is a feature of *Plasmodium cynomolgi* B strain infection. **a** Reticulocyte Concentration of five rhesus macaques are shown prior to infection, during the acute primary, and after the peak of parasitemia. **b** Reticulocyte dynamics in relation to parasitemia for up to 100 days after inoculation are depicted. *Orange bars* indicate when peripheral blood and bone marrow aspirates were collected for retrospective systems biology analysis. **c** The relationship between hemoglobin and reticulocyte concentrations are shown for up to 100 days after inoculation. Statistical analysis was performed by a Friedman's Test with Dunn's multiple comparison post-hoc for *Panel* **a**; ^*^p < 0.05, *ns* not significant

Reticulocytes were enumerated to monitor the clinical situation, the animals’ attempts to recover from anaemia, and as a peripheral indicator of erythropoiesis in the bone marrow. Under homeostatic conditions, reticulocyte release will coincide with decreases in peripheral haemoglobin concentration to compensate for the loss of RBCs in the periphery. Thus, reticulocyte concentrations should increase during the acute, primary infection period and begin to return to pre-infection levels after the peak of parasitaemia. Contrary to this hypothesis, the reticulocyte concentrations did not increase from pre-infection levels until the post-peak parasitaemic phase when the animals were beginning to show signs of recovery (Figs. 3a, b, c). Curiously, there was a small increase in reticulocyte concentrations between approximately days 8 and 15 post-inoculation for all animals except RFv13 (the animal with the most severe disease), followed by an immediate decrease back to pre-infection levels whenever parasitaemia began to increase exponentially (Fig. 3b, c).

### Thrombocytopaenia developed during the primary blood-stage infections

**Fig. 4:**
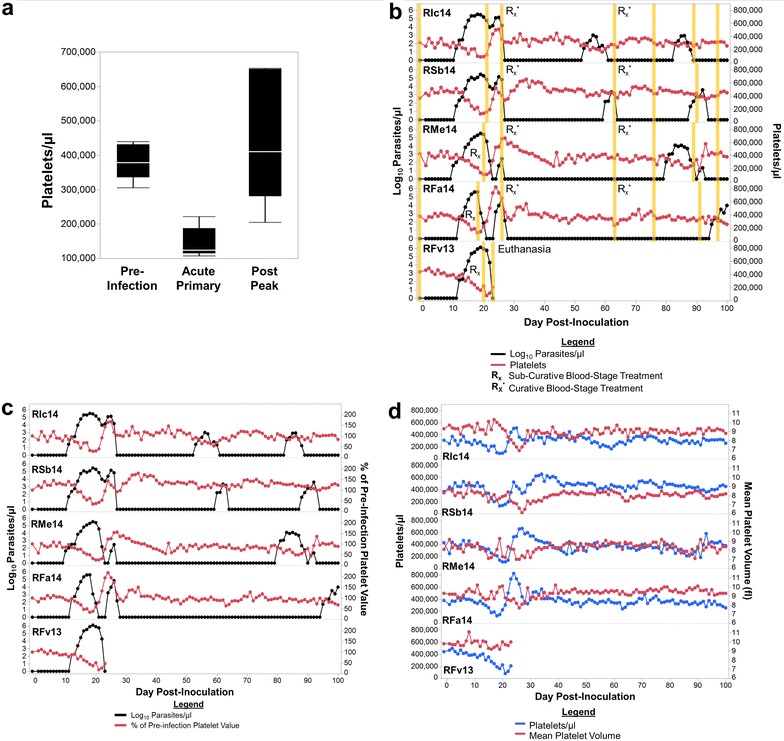
*Plasmodium cynomolgi* B strain infection results in thrombocytopenia during the acute primary infection. **a** The mean platelet concentrations before infection, during the acute primary, and post peak of parasitemia are shown. **b** Parasitemia in relation to platelet concentration is shown for each macaque. *Orange bars* indicate when peripheral blood and bone marrow aspirates were collected for retrospective systems biology analysis. **c** Parasitemia in relation to the percent of pre-infection platelet values is shown. **d** The mean platelet volume in relation to the platelet concentration is shown. Statistical analysis was performed by a Friedman's Test with Dunn's multiple comparison post-hoc for *panel* **a** and no statistical significance was found

Thrombocytopaenia, a clinical condition that is the result of reduced number of platelets in the blood, is a common haematological finding during malaria, but its specific role in pathogenesis has been highly debated [14, 44–48]. Indeed, thrombocytopaenia (<150,000 platelets/µl) was observed in all macaques (Fig. 4a, b). Despite the decrease below a clinically relevant threshold, the drop in platelet concentration was not statistically significant from pre-infection values (*p* > 0.05), and furthermore, severe thrombocytopaenia (<50,000 platelets/µl) was not observed (Fig. 4a, b). Thrombocytopaenia was most pronounced at day 20, near the peak of parasitaemia, where the peripheral platelet concentrations were approximately 38 % of the baseline levels (Fig. 4b, c). The recovery of platelets coincided with a decrease in parasitaemia between days 20 and 26, irrespective of the administration of sub-curative blood-stage treatment or self-control of the infections (Fig. 4b). Notably, platelet concentration in the periphery increased to higher than pre-infection values before stabilizing (Fig. 4b, c).

**Fig. 5:**
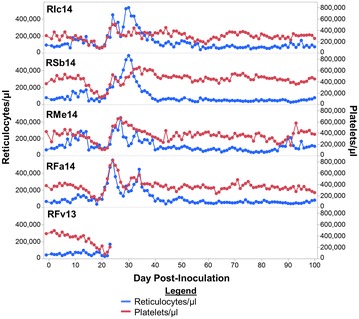
Reticulocyte and platelet concentrations recover at a similar rate after the acute primary infection. The reticulocyte and platelet concentration are shown for up to 100 days after infection of rhesus macaques with *P. cynomolgi* B strain

Thrombocytopaenia is thought to be the result of a variety of mechanisms, including the binding of platelets to infected RBCs, removal of platelets by phagocytic cells and sequestration of the platelets in the microvasculature and organs [14, 16, 37]. Here, a decrease in mean platelet volume (MPV) was noted whenever platelet concentrations were beginning to return to pre-infection levels, between days 20 and 30 post-inoculation (Fig. 4d). Alterations in MPV could indicate that platelet production by megakaryocytes in the bone marrow was disrupted, providing another mechanism that could potentially contribute to thrombocytopaenia. In addition to impaired production of platelets, a lower MPV could be caused by low platelet concentrations. However, the current data do not support this explanation, because as platelet concentrations returned to pre-infection levels the MPV decreased (Fig. 4d). Furthermore, MPV and platelet concentration were inversely correlated (Spearman ρ = −0.3785, p < 0.0358) (Fig. 4d). To further evaluate the hypothesis that megakaryocyte function could be compromised during cynomolgi malaria, the dynamics of reticulocyte release and platelet recovery were examined. If platelet and/or reticulocyte production were compromised due to altered bone marrow physiology, then the recovery of these cell types in the periphery may overlap. Indeed, the increase of platelet concentrations after the primary blood-stage infections coincided with increases in peripheral reticulocyte concentrations (Fig. 5).

### Relapses did not result in significant changes in clinical parameters

**Fig. 6:**
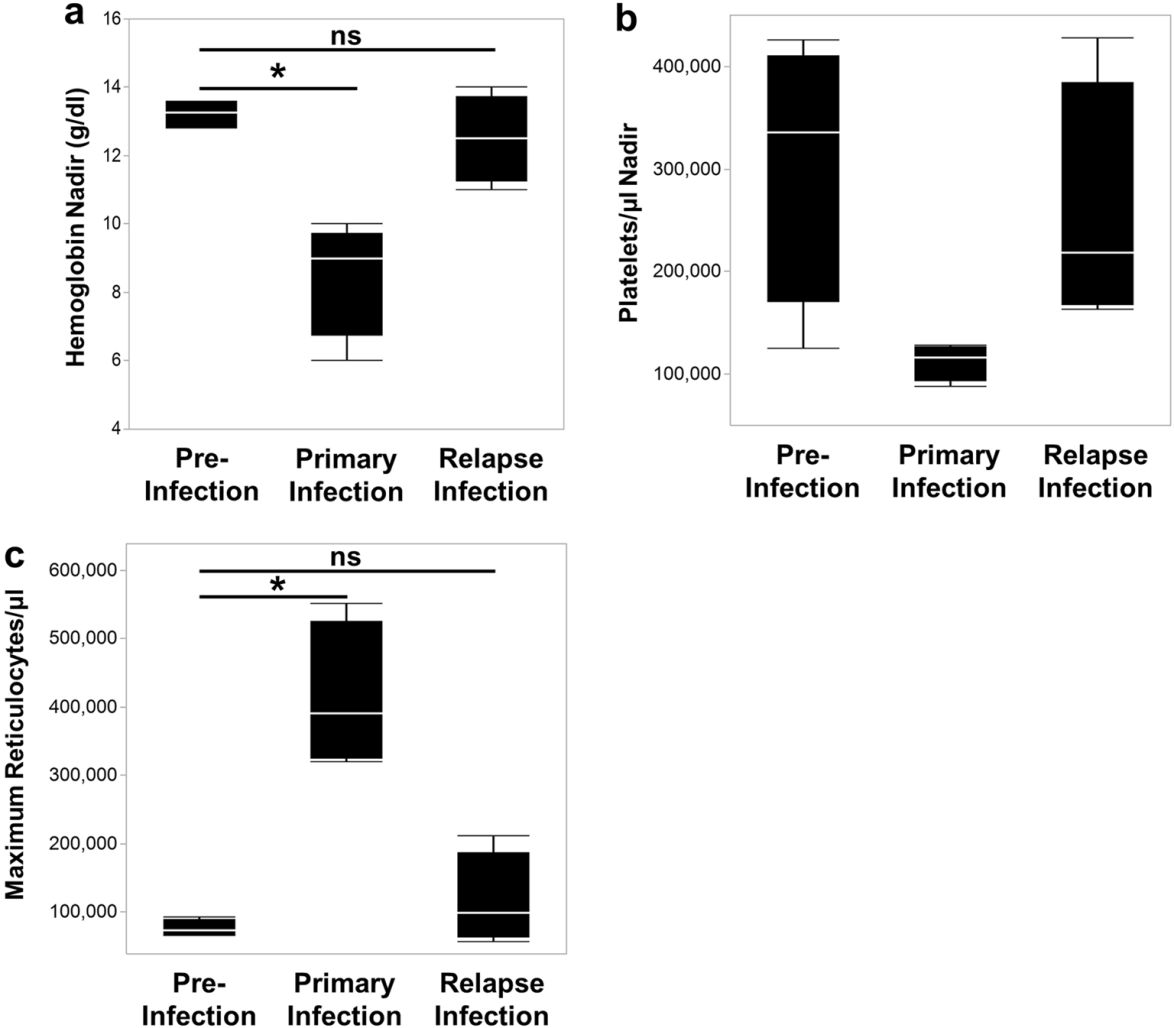
Homologous relapses induce minimal, if any, changes in clinical parameters from pre-infection values. The average of haemoglobin concentrations (**a**), platelet nadirs (**b**), and maximum number of reticulocytes (**c**) for RMe14, RFa14, RIc14, and RSb14 prior to infection, during the primary infection, and for relapses are shown. Statistical significance was assessed by a Friedman's Test with Dunn's multiple comparison post hoc analysis. pvalues less than 0.05 were considered significant; ^*^p < 0.05; *ns* not significant

One of the primary goals of this study was to better understand the clinical aspects of relapses compared to primary blood-stage infections. Analyses focused on the first relapses, with comparisons across the four animals that survived the primary infection. Importantly, significant changes were not observed in parasite burden (Fig. 1b), haemoglobin (Fig. 2b), reticulocyte (Fig. 3b), or platelet concentrations (Fig. 4b) during the second relapses compared to the first. Relapses did not result in the same changes in clinical parameters as the primary infections and did not differ significantly from pre-infection values (Fig. 6a, b, c).

RMe14, the macaque with the highest parasitaemia during a relapse, was the only animal that developed minor alterations in haemoglobin, platelet and reticulocyte concentrations during relapses (Figs. 2b, 3b, 4b). Specifically, the haemoglobin level for this animal dropped from 14 g/dl, recorded 7 days before the peak of the relapse parasitaemia, to 11 g/dl, making it mildly anaemic (Fig. 2b). Unlike during the period of the primary blood-stage infection, the drop in haemoglobin concentration resulted in an increase in the peripheral reticulocyte concentration, suggesting that the mechanism(s) that prevented the compensatory release of reticulocytes to compensate for alterations in haemoglobin concentration during the primary infection did not occur during relapse infections, likely due to the controlled parasite burden (Fig. 3b). Platelet concentration also dropped sporadically during relapses for this animal (Fig. 4b).

### Clinical presentations ranged from non-severe to lethal

A variety of clinical phenotypes were observed in this study, which could be distinguished as non-severe, severe and lethal (Table 2). RIc14 and RSb14 were able to control the primary infection without sub-curative blood-stage treatment and displayed mild thrombocytopaenia and moderate levels of anaemia. Thus, these two animals were classified as non-severe. Contrastingly, RFa14, RMe14 and RFv13 required sub-curative treatment during the primary blood-stage infection due to a variety of adverse clinical signs. RFa14 presented with a unique phenotype characterized by a drop in haemoglobin to approximately 11.2 g/dl and platelet concentration to approximately 125,000 platelets/µl in the peripheral blood early during the primary infection, and uniquely, petechiae were noticed on the trunk of this animal.

This profile was consistent with that reported with a *P. coatneyi* infection in a rhesus macaque, where the animal progressed to very severe disease due to disseminated intravascular coagulopathy [49]. Due to the poor prognosis associated with these indications, RFa14 was sub-curatively treated and classified as severe. Contrastingly, RMe14 and RFv13 developed severe anaemia (<7 g/dl) during the primary infection (Fig. 2b). In response to this presentation, sub-curative blood-stage treatment was administered and whole blood transfusions performed for both of these animals. RMe14 recovered and was categorized as having a severe clinical phenotype, but RFv13 succumbed to the infection as noted above despite this intervention, resulting in the additional classification as a lethal clinical phenotype.

### Parasitaemia and the lack of an increase in reticulocytes during the initial phase of the primary infection distinguish the lethal clinical phenotype

**Fig. 7:**
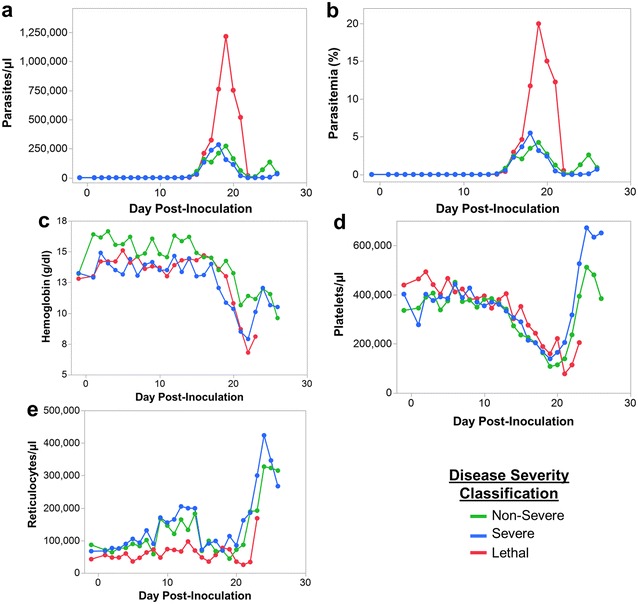
Higher parasitaemia and less reticulocytes during early infection distinguish the lethal cynomolgi malaria infection. Parasitaemia (**a**, **b**), haemoglobin (**c**), reticulocyte (**d**), and platelet (**e**) kinetics are shown comparing the three different cynomolgi infection clinical phenotypes. The average of the two non-severe and two severe animals were used for each point on the graph; since the lethal phenotype is unique, a single animal’s values were used

The contribution of parasite burden to each clinical phenotype was evaluated. The peak parasitaemia in the non-severe animals was 273,509 and 305,636 (mean = 289,573) parasites/µl whereas the two severe animals that survived had parasitaemias of 309,365 and 379,140 (mean = 344,452) parasites/µl during the primary infection (Figs. 1b, 7a). Using this measure, the parasitaemia was not strikingly different between the non-severe and severe clinical phenotypes. Next, the peripheral parasitaemias were analysed as a percentage, or the number of infected RBCs out of the total RBCs. Using this approach, there was possibly a minor difference between the two phenotypes. Specifically, the non-severe animals had parasitaemias of 4 and 5 % whereas the severe animals had parasitaemias of 6.3 and 7.8 %, prior to administration of the sub-curative blood-stage treatments (Fig. 7b). The animal that succumbed to the infection developed a maximum parasitaemia of approximately 1,200,000 parasites/µl and showed a 19.5 % parasitaemia prior to clinical intervention, which was approximately four-fold higher than observed in the surviving animals (Fig. 7a, b).

Unexpectedly, one other clinical feature in addition to parasite burden was identified that distinguished the lethal from the non-severe and severe phenotypes. Even though the haemoglobin concentrations and platelet kinetics for the non-severe, severe and lethal phenotypes were all similar during the primary blood-stage infections (Fig. 7c, d), the reticulocyte kinetics for the lethal infection (RFv13) were not the same as for the other two phenotypes (Fig. 7e). Specifically, as noted above, the peripheral reticulocyte concentrations in this animal did not show a modest increase during the patent blood-stage infection between days 10–15 post-inoculation, in comparison to the other animals where a modest, short-term increase was evident (Fig. 7e). This difference in reticulocyte kinetics could indicate that this and possibly other early host responses may influence and be a predictor of clinical complications and the potential course of the disease state or recovery processes.

## Discussion

This 100-day experiment was designed to study the clinical and parasitological attributes of the primary blood-stage and relapse infections caused by *P. cynomolgi* in rhesus macaques and set the stage for future, comprehensive studies using this model to understand the underlying mechanisms of immunity, pathogenesis and disease. Highly resolved kinetics of core haematological parameters were developed after inoculation with infectious B strain sporozoites, and specifically, reticulocyte, platelet, haemoglobin, MPV, and MCV dynamics are presented here. This study expands upon previous rhesus infection studies with the *P. cynomolgi* B strain, which primarily monitored parasitological data but did not assess clinical parameters on a daily basis [17, 32]. The compiled clinical and parasitological datasets generated here provide a glimpse into the daily dynamics of malaria beyond what is typically feasible in humans. It is anticipated that data generated in this experiment (all of which are being shared publicly—see “Methods” section and Additional file 1) will aid the development and testing of hypotheses by the research community whether for clinical studies or continued investigations using *P. cynomolgi* as a model.

Anaemia ranged from mild to severe during the primary infections in this study and appears to be the result of multiple processes. First, it is noteworthy that haemoglobin decreased to the lowest levels after parasitaemia was reduced, whether from the natural control of the infection by the macaque or drug treatment. This data demonstrates that the loss of RBCs due to parasitism is not the sole reason for anaemia in this model. Indeed, other non-human primate malaria models [43], rodent malaria models [13] and humans [50, 51] show a similar kinetic where anaemia is worse after the peripheral parasitaemia has decreased. This phenomenon is attributed to the simultaneous removal of iRBCs and uninfected RBCs [52, 53], which has also been modelled mathematically to arrive at similar conclusions [50]. Secondly, the normal, timely replenishment of circulating RBCs by the release of reticulocytes from the bone marrow was disrupted, as shown previously with *P. coatneyi* infection of rhesus macaques [43]. Thus, the disruption of compensatory bone marrow mechanisms with the effective release and/or production of reticulocytes from the bone marrow is likewise apparent in the *P. cynomolgi*-rhesus macaque model during the acute, primary infection. For each of these species, which model *P. falciparum* and *P. vivax*, respectively, the disruption of normal bone marrow physiology could be potentially due to ongoing immunological responses against the parasite or parasite byproducts, such as haemozoin, that are released as the parasites multiply in the blood [13, 54–56]. In agreement as shown here, there was an initial increase of reticulocytes early during the primary infections and these cells returned to baseline levels when the parasitaemia was increasing exponentially in the blood. This supports the view that the disruption of the normal compensatory mechanisms is dependent on parasite levels. Future studies will be needed to better understand the host-pathogen interactions that occur during the primary infection and how they contribute to malarial anaemia in this model.

The relative contribution of thrombocytopaenia to disease outcome during malaria remains controversial and also not well understood [14, 44, 47]. In this study, all animals developed mild thrombocytopaenia irrespective of their clinical presentations, and thus, thrombocytopaenia was not viewed as an indicator of disease severity or outcome. Prior evidence suggests that thrombocytopaenia could be due to removal of platelets by splenic macrophages during infection [16], whereas other models suggest that platelets form clumps with iRBCs [57] or become sequestered in the microvasculature by adhering to activated endothelial cells [58]. Interestingly, published experiments with the *P. cynomolgi* B strain have demonstrated that thrombocytopaenia occurs in both spleen-intact and splenectomized macaques, suggesting that there are other contributing factors in the development of this complication aside from processes associated with this organ [59]. In agreement, the data from this study indicates that platelet production may have been impaired since the MPV was decreased as the number of platelets rebounded after the peak of parasitaemia. Previous studies with malaria patients have reported changes in MPV during malaria [16, 60]. The alteration in MPV may indicate disruption of megakaryocyte function, and thus, platelet production. In further support of this hypothesis, both reticulocytes and platelets returned to pre-infection levels together, and since both of these cell types originate from the bone marrow, the simultaneous return to normal levels could be explained by the restoration of normal bone marrow physiology. Overall, these data suggest that the disruption of normal bone marrow physiology during acute malaria may also lead to impaired platelet production and contribute to thrombocytopaenia. Future studies should explore this hypothesis further and aim to understand if thrombocytopaenia is, in part, a bystander effect of bone marrow dysfunction.

It is important to recognize that macaques infected with *P. cynomolgi* presented with inter-individual differences in disease severity, similar to humans. In RFv13, a very high parasitaemia (19.5 % parasitaemia), appeared to contribute to the lethality since this parasitaemia clearly distinguished this animal from the two non-severe (RSb14 and RIc14) and two severe (RMe14 and RFa14) clinical presentations. These phenotypes did not clearly stratify away from each other based on parasitological profiles alone, indicating that while parasitaemia may play a role in severe disease, it is not solely responsible for the observed differences in clinical presentation and other pathological processes may come into play. This is reminiscent of human malaria where individuals can progress to severe malaria regardless of parasitaemia [61–63]. Curiously, unlike the four surviving macaques, RFv13 did not show a modest increase in reticulocytes during the beginning of the primary infection. In future studies, it would be useful to evaluate this finding and other potential indicators of the progression of infections and how these early responses to blood-stage infections influence pathogenesis and clinical presentation.

Schmidt [32] demonstrated that relapse frequencies in the rhesus macaque differ based on the number of *P. cynomolgi* B strain sporozoites inoculated, and based on this evidence, some investigators have used 1 × 10^6^ sporozoites to generate early, frequent and uniform relapse patterns across individuals for screening anti-hypnozoite drugs [19]. In contrast, only about 2000 sporozoites were inoculated in the current study, aiming for primary infections followed by more natural patterns of relapse. While this is still higher than what is suspected to be naturally injected by a mosquito [64, 65], 2000 sporozoites best ensured at least one or two relapses in all animals during the course of this study. In fact, the average number of relapses observed was 1.5. This number may have been higher if the animals had not been cured as a group after the fifth specimen collection. Nevertheless, the observed relapse patterns reported here are similar to what would be expected with *P. vivax* infections with tropical strains of this parasite [7, 66, 67]. A follow-up, iterative experiment was designed to monitor the natural relapse patterns, without such treatment plans, and to monitor gametocytes during the relapse periods more carefully.

Understanding, preventing and treating relapses caused by *P. vivax*, and also *P. ovale,* remain key challenges in today’s malaria eradication efforts, especially if asymptomatic carriers remain infectious to mosquitoes. Critically, the relative impact of primary *versus* relapse clinical malaria presentations has remained undefined in a controlled, experimental model system and direct evidence from human studies is not available because of the challenges distinguishing the two in human cases without a well-controlled study design [68–71]. Here, each *bona fide* relapse, subsequent to blood-stage treatment and in the absence of re-infections, resulted in significantly lower parasitaemias compared to the primary infections and minimal, if any, changes in clinical parameters to indicate illness. This suggests that immunity, or potentially other undefined mechanisms, during the primary infection, led to controlled relapses characterized by reduced parasitaemias and lack of clinical complications. Whenever minor alterations in clinical parameters were observed during a relapse (e.g., minor drop in haemoglobin levels from 14 to 11 g/dl), as was the case for RMe14, these alterations resolved in a controlled, non-pathological manner, without the need for clinical support. The lack of clinical illness during relapses was not necessarily expected, especially in animals that only experienced a single primary blood-stage infection, since previous reports have concluded, through mathematical and statistical modelling of data collected from holo-endemic areas that relapses are responsible for up to 96 % of blood-stage *P. vivax* infections [11, 72]. Thus, this experiment draws light to the question “What percentage of relapses actually cause clinical malaria?” Relapses have been a major concern due to their role in causing possible repeated bouts of illness and because they can be a source of infectious gametocytes to maintain transmission. The data presented here support the hypothesis that relapses may not necessarily be the main cause of clinical vivax or ovale malaria cases. This warrants further study to understand the clinical outcome of primary infections compared to relapses, in addition to transmissibility questions, caused by homologous or heterologous strains as would also be anticipated in malaria-endemic regions [71, 73, 74].

In a previous study, rhesus macaques infected with *P. cynomolgi* were treated soon after infected RBCs were detected, and the parasitaemia of the first relapses appeared to be higher than subsequent relapses [19]. The clinical status of the macaques during relapses was not presented, however, so it is unknown if the clinical profile of the relapses differed from the primary infections. In contrast, the parasitaemia in the current study was allowed to persist during the primary infections, since a goal was to understand the course of the clinical presentations. Interestingly, with this approach, the first and second relapses had similar parasitological and clinical attributes, and the difference in parasitaemia between the primary infection and relapses was substantial. These data highlight the point that the timing of the experimental treatment of blood-stage infections may affect the later presentation of relapses, and could in turn influence whether the relapse parasitaemia and illness are suppressed, or not. Future studies should evaluate if the timing of blood-stage treatment affects the development of immunity against the parasite.

As shown by this research team and others using this model system, relapse infections can be definitively produced under controlled experimental conditions [19, 32], in the absence of new sporozoite-initiated infections, and they can be distinguished from blood-stage recrudescences through the administration of curative blood-stage treatments. This creates a clear ‘window’ of time after the administration of curative treatment of blood-stage infections, during which time the activation of hypnozoites and appearance of relapse parasites can be anticipated and studied. While a challenging prospect, it is during this period that the identification of blood-based, metabolic biomarkers of liver-stage forms may 1 day become a reality, paving the way towards diagnostic tools and curative liver-stage treatments [75].

## Conclusions

The *P. cynomolgi*-rhesus macaque model is invaluable for malaria research, particularly with regard to the direct study of both primary and relapse infections and pathophysiological mechanisms. This study uniquely provides a day-to-day clinical perspective in haematological changes during longitudinal infections, and sets the stage for systems biology investigations based on data from a few individuals [76, 77]. Here, all five subjects showed different degrees of clinical severity. It is anticipated that future analyses (immunological, biochemical, etc.) using the blood and bone marrow specimens collected from this experiment, with the integration of such data with the fundamental information presented here, will lead to the identification of possible mechanisms and an improved understanding of anaemia, thrombocytopaenia and relapses. Notably, the current data have provided insight into relapse biology by demonstrating that *bona fide* relapses did not result in signs of clinical malaria. This was not necessarily expected and is important to consider when assessing both the clinical and epidemiological impact of relapses caused by *P. vivax* and *P. ovale*.

## Additional file

10.1186/s12936-016-1480-6 Haematological and parasitological data of *Plasmodium cynomolgi* B strain infections in rhesus macaques for up to 100 days. The data used in this study are contained within this file with appropriate metadata.

## Abbreviations

CBC: complete blood count
CDC: Centers for Disease Control and Prevention
YNPRC: Yerkes National Primate Research Center
IACUC: Institutional Animal Care and Use Committee
FCS: fetal calf serum
iRBC: infected red blood cell
RBC: red blood cell
LIMS: Laboratory Information Management System
SOP: standard operating procedure
MCV: mean corpuscular volume
MPV: mean platelet volume
WBC: white blood cell
WHO: World Health Organization
